# An Accurate Estimate of the Free Energy and Phase Diagram of All-DNA Bulk Fluids

**DOI:** 10.3390/polym10040447

**Published:** 2018-04-16

**Authors:** Emanuele Locatelli, Lorenzo Rovigatti

**Affiliations:** 1 Faculty of Physics, University of Vienna, Boltzmanngasse 5, A-1090 Vienna, Austria; 2 CNR-ISC, Uos Sapienza, Piazzale A. Moro 2, 00185 Roma, Italy; lorenzo.rovigatti@uniroma1.it; 3 Department of Physics, Sapienza Università di Roma, Piazzale A. Moro 2, 00185 Roma, Italy

**Keywords:** DNA, DNA nanotechnology, patchy particles, Wertheim theory, thermodynamic integration, phase coexistence

## Abstract

We present a numerical study in which large-scale bulk simulations of self-assembled DNA constructs have been carried out with a realistic coarse-grained model. The investigation aims at obtaining a precise, albeit numerically demanding, estimate of the free energy for such systems. We then, in turn, use these accurate results to validate a recently proposed theoretical approach that builds on a liquid-state theory, the Wertheim theory, to compute the phase diagram of all-DNA fluids. This hybrid theoretical/numerical approach, based on the lowest-order virial expansion and on a nearest-neighbor DNA model, can provide, in an undemanding way, a parameter-free thermodynamic description of DNA associating fluids that is in semi-quantitative agreement with experiments. We show that the predictions of the scheme are as accurate as those obtained with more sophisticated methods. We also demonstrate the flexibility of the approach by incorporating non-trivial additional contributions that go beyond the nearest-neighbor model to compute the DNA hybridization free energy.

## 1. Introduction

The most prominent function of DNA is to store the genetic information of all living organisms. However, the main biophysical features that allow DNA to fulfil its role, namely, the extremely regular structure of its double-stranded form and the outstanding specificity of its single-stranded form, make it a great addition to the toolboxes of nanotechnology and materials science [1,2]. The usage of DNA in the latter field is extremely variegated, as it can be used not only as a link to connect nano- or micro-particles [3,4], but also to synthesize all-DNA materials. In both cases, the resulting materials can be made either ordered or disordered [5,6,7,8]. An important factor that contributes to the complexity of DNA self-assembly is its polymeric nature [9,10,11,12,13,14]. Biologically, it can be found in linear or circular form [15,16]; the thermodynamical [17,18] and topological [19,20,21,22] properties of DNA as a biopolymer have been the object of increasing interest in the recent past. On the other side, DNA has been used to synthesize polymeric materials such as dendrimers [23,24] or hydrogels [25,26].

The strategy devised to synthesize all-DNA materials is based on the concept of hierarchical or multi-step self-assembly: short (usually 10–100 nucleotides) DNA single strands are designed to self-assemble into a well-defined object at intermediate temperature *T*. As *T* is lowered, these DNA constructs start to aggregate and form higher-order finite structures or even bulk materials [1]. Because the melting temperature of DNA is highly dependent on the specific strand sequence and its length, the temperature at which the self-assembly of the constructs and the formation of inter-construct bonds occur can be tuned independently. Therefore, it is common to use sequences of length 4–10 for the inter-construct bonding and of length 15–30 for the constructs themselves. Sequences designed to follow this principle have been used to synthesize crystals [7], gels [8] and reentrant gels [27]. Most of the time, these synthetic sequences have to be chosen carefully and through a tedious and expensive trial-and-error procedure. Indeed, from a theoretical standpoint, there are two-state thermodynamic descriptions of DNA, the “nearest-neighbour” models, that make it easy to estimate the melting temperature (and even secondary structure) of DNA sequences [28]. However, there is no straightforward way of predicting the actual phase behaviour of complicated DNA systems. For all-DNA constructs that interact and bind through a small number of short sticky ends, some progress has been made by employing a well-known liquid-state theory, the Wertheim thermodynamic perturbation theory (WTPT) [29,30], complemented by detailed simulations of a realistic DNA model [31]. The output of these calculations is the free energy of the system, from which it is possible to estimate the thermodynamic stability of the different phases as a function of temperature and density (or concentration). It has been shown that this hybrid theoretical/numerical approach is able to semi-quantitatively (and, in some cases, quantitatively) reproduce experimental results. However, the method developed in [31] rests on some assumptions and approximations that were not fully controlled and require a more stringent validation in light of their sheer simplicity. For example, rather than requiring cumbersome simulations of bulk systems, the approach of [31] efficiently evaluates the free energy by employing results from two-body simulations as inputs for a second-order virial expansion.

In this paper we employ large-scale molecular dynamics simulations to complement the WTPT and compute the phase behaviour of all-DNA systems with higher accuracy, in order to more strictly validate the method introduced in [31]. In particular, we run extensive bulk simulations to calculate the free energy with unprecedented accuracy by considering a third-order virial expansion and by thermodynamic integration. We also show that the method can be readily extended by considering additional contributions. We further improve the agreement between theory and experiment by assessing, for the first time, the electrostatic effect due to the presence of long tails on the hybridization of the sticky ends [32] and hence on the nanostar phase behaviour.

The paper is organized as follows: In Section 2, after discussing the estimation of the Helmoltz free energy within the WTPT framework, we briefly discuss the determination of critical points and phase coexistence; we further briefly review the numerical models. In Section 3, we detail all the results obtained. First, we characterize the structure of the fluid (Section 3.1); we then examine and comment on the data obtained from the numerical simulation for the pressure as a function of the concentration at different salt concentrations and different temperatures (Section 3.2). Last, we compute the critical points for trivalent and tetravalent nanostars, comparing the results with previous estimates (Section 3.3).

## 2. Methods

### 2.1. DNA Sequences

**Figure 1:**
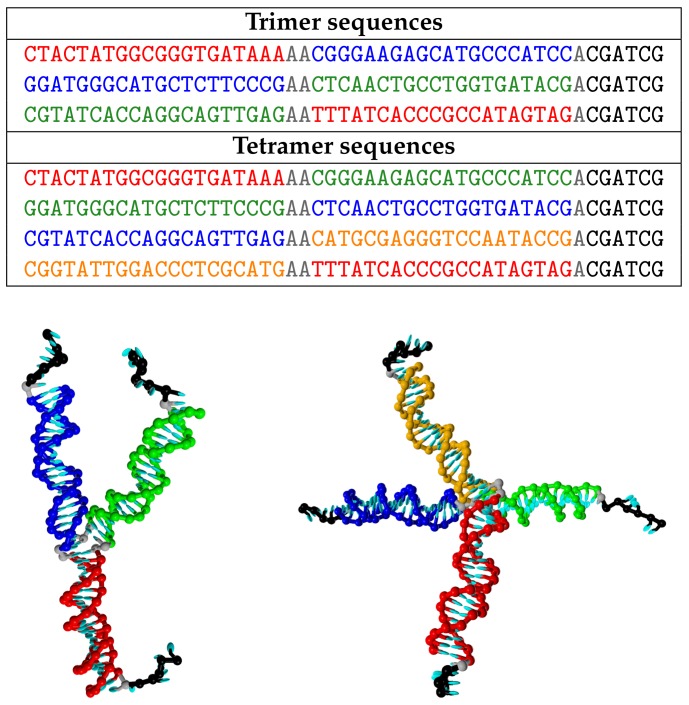
(**Top**) Strand sequences for the DNA constructs used in this work. Spacers (nucleotides that add flexibility to the structure but are not designed to pair up) are coloured in grey. Non-grey nucleotides that are part of complementary sequences share the same colour. (**Bottom**) Simulation snapshots of a trimer and a tetramer, coloured according to the same color convention.

We consider systems made up of all-DNA nanostars, that is, branched polymers with *f* arms. These DNA constructs are composed of *f* single strands with specifically designed sequences (see Figure 1). The sequences used are the same as those reported in previous experimental works [8,33,34].

We focus on trivalent (trimers, f=3) and tetravalent (tetramers, f=4) constructs. In both cases, each of the *f* strands are composed of 49 nucleotides. Each sequence is divided into two central regions (20 nucleotides each), and a third, shorter region (6 nucleotides). The two central regions are designed to form the stiff double-stranded sections of the arms; they are separated by two unpaired nucleotides, also called spacers, that provide flexibility at the centre of the construct. The third region is identical in all four sequences; one spacer is present before the sticky end, also providing flexibility. The final sequence is self-complementary and thus allows for inter-tetramer bonding. The difference in length between the double-stranded arms and the sticky ends provides a separation between the temperatures at which bonds and arms melt. This allows for a temperature-controlled hierarchical self-assembly scenario, where the *f* single strands can spontaneously assemble at intermediate temperatures and form stable constructs before bonds can be formed. Therefore, there is a range of temperatures in which the construct is stable and can represent an all-DNA experimentally accessible realization of a patchy particle [8,27,35]. As will be also mentioned later, in this work we scramble the sticky end sequences so that no bonding is possible. Such expedient provides, in the case of DNA constructs, the *reference fluid*.

### 2.2. Numerical Methods

We perform simulations of DNA nanostars modeled with oxDNA2 [36], a DNA model coarse-grained at the level of single nucleotides. The interaction forms and parameters in oxDNA2 are chosen to reproduce structural and thermodynamical properties of both single- and double-stranded DNA molecules in B-form. The interactions between nucleotides, modeled as rigid bodies, account for excluded volume, electrostatic repulsion between the negatively charged backbones, backbone connectivity, Watson–Crick hydrogen bonding, stacking, cross-stacking and coaxial stacking. The interaction parameters have been adjusted in order to be consistent with experimental data on the structure and thermodynamics of DNA [37,38]. The electrostatic repulsion is provided by a Yukawa term characterized by a screening length that is an increasing function of *T* and a decreasing function of the salt concentration *S* [38]. Multivalent salts are not supported by the model.

We ran large-scale simulations (250 purely repulsive trimers or tetramers for a total of 36,750 or 49,000 nucleotides, respectively) at eight different densities, three salt concentrations and three temperatures (15 and 20 ${}^{\circ }$C for tetramers and $20{\;}^{\circ }$C for trimers). We performed molecular dynamics simulations in the NVT ensemble using a Brownian thermostat [39]. The equations of motion were integrated with the velocity Verlet algorithm with a timestep of Δt=0.001. We performed these simulations on NVIDIA GTX1080 GPUs [40]. We equilibrated for up to $10^{7}$ MD steps and, in order to calculate the equation of state with adequate precision, performed production runs for ∼10${}^{9}$ MD steps (2–3 weeks of single-GPU wall time, depending on the salt concentration). The pressure was computed by using the *molecular* definition of the virial [41].

### 2.3. Theoretical Framework

We combine the WTPT with an accurate mass-action law describing DNA binding. In a pure, one-species system, within the representation of the Helmoltz free energy, the thermodynamic quantities can be expressed as functions of the temperature *T* and the density ρ. In the WTPT, the Helmholtz free energy per particle for DNA nanostars of valence f=4 (tetramers) or f=3 (trimers) can be written as follows [31,42]:

(1) $$\beta \bar{f}\left(T,\rho \right)\equiv \beta \frac{F(T,\rho )}{N}=\beta f_{\mathrm{ref}}\left(T,\rho \right)+\beta f_{b}\left(T,\rho \right),$$

where $\beta =1/k_{B}T$, $k_{B}$ is the Boltzmann constant. Following the standard notation, $\beta f_{\mathrm{ref}}\left(T,\rho \right)$ is the free energy per particle of the reference system, that is, the system in which particles are not decorated with bonding sites, and $\beta f_{b}\left(T,\rho \right)$ is the free energy per particle associated to the bond formation. The former term, in the case of DNA nanostars, is evaluated by considering a system in which the sticky end sequences are scrambled in such a way that Watson–Crick pairing does not occur and no inter-star bond can form; the residual interaction is purely repulsive. $\beta f_{\mathrm{ref}}$ itself is given by the two contributions:

(2) $$\beta f_{\mathrm{ref}}\left(T,\rho \right)=\beta f_{\mathrm{id}}\left(T,\rho \right)+\beta f_{\mathrm{ex}}\left(T,\rho \right),$$

where the ideal-gas free-energy density is given by $\beta f_{\mathrm{id}}\left(T,\rho \right)=\ln\left(v_{0}\rho \right)-1$; here $v_{0}$ is a reference volume whose value has no effect on the derivatives of the free energy. Because WPTP is often applied to systems with hard-core excluded volume interactions, the free energy of the reference fluid is usually approximated with the Carnahan–Starling expression. Here, however, we deal with a system for which there are unknown theoretical estimates. In [31], a simple virial expansion truncated at the second order was adopted for the sake of simplicity and numerical efficiency. Here we extensively check the quality of the approximation by providing more accurate estimates of the reference free energy. More specifically, we compute the excess free energy per particle following two standard routes: thermodynamic integration (TI) and the virial expansion truncated at the third order.

The standard path of thermodynamic integration consists of computing the free energy from a known reference state through integration of the equation of state as

(3) $$\beta f_{\mathrm{ref}}\left(T,\rho \right)=\beta f_{\mathrm{id}}\left(T,\rho \right)+\beta f_{0}\left(T,\rho \right)+\int_{0}^{\rho }\frac{\beta P\left(\rho^{\prime }\right)-\rho^{\prime }}{\rho^{\prime 2}}d\rho^{\prime },$$

where *P* is the pressure of the reference fluid, which is the main output of the bulk simulations. We evaluate *P* at different densities ρ, temperatures and salt concentrations. Because there is essentially no attraction between the DNA nanostars in the reference system, we expect the pressure to be a convex well-behaving function of the density. Therefore, for each (T,S) pair we can span almost 3 orders of magnitude in density by simulating just eight state points. Once *P* is calculated with sufficient precision, we interpolate and integrate it analytically within the range of densities considered. The pressure measurements done in the bulk, large-scale numerical simulations performed can be also used to improve the virial expansion, which was previously limited at the second order [31]. Within the virial expansion, the pressure as a function of density reads

(4) $$\beta P=\rho +B_{2}\rho^{2}+B_{3}\rho^{3},$$

where $B_{2}=B_{2}\left(T\right)$ is the second virial coefficient:

(5) $$B_{2}\left(T\right)=-\frac{1}{2}\int_{0}^{\infty }4\pi r^{2}\left(\exp\left(-\frac{V(r)}{k_{B}T}\right)-1\right)dr$$

computed at different salt concentrations; V(r) is here the effective intra-molecular pair potential. We obtain the third virial coefficient through a fitting procedure; then we write the excess free energy as in [31], employing the virial expansion:

(6) $$\beta f_{\mathrm{ex}}\left(T,\rho \right)=B_{2}\left(T\right)\rho +\frac{B_{3}\left(T\right)}{2}\rho^{2}.$$

Finally, as all bonds are identical, the bonding free energy per particle is given by the following [43]:

(7) $$\beta f_{b}\left(T,\rho \right)=f\left(\ln\left(1-p_{b}\left(T,\rho \right)\right)+\frac{1}{2}p_{b}\left(T,\rho \right)\right),$$

where $p_{b}$ is the fraction of formed bonds. The latter, which is a function of *T* and ρ, can be evaluated via a law of mass action, yielding

(8) $$p_{b}\left(T,\rho \right)=1-\frac{-1+\sqrt{1+4\Delta f\rho }}{2\Delta f\rho },$$

where Δ is linked to the free-energy difference between bonded and non-bonded pairs of sticky ends. Following [28,44],

(9) $$\Delta \equiv v_{b}\exp\left(-\beta \Delta G\right)=v_{b}\exp\left(-\frac{\Delta H-(\Delta S_{\mathrm{nosalt}}+\Delta S_{\mathrm{salt}})T}{k_{B}T}\right),$$

where $v_{b}=1.6606$ nm${}^{3}$ is the reference volume of the non-bonded single strands [45], ΔH=−54,000 cal is the enthalpy gain upon bonding, and $\Delta S_{\mathrm{nosalt}}=-151.99$ cal/K and $\Delta S_{\mathrm{salt}}=0.368\cdot \left(L_{\mathrm{DNA}}-1\right)\cdot \ln\left(S\right)$ cal/K are the salt-independent and -dependent entropy variations again upon bonding, respectively. These quantities refer to the sticky end sequences considered here, for which $L_{\mathrm{DNA}}=6$ (see Appendix A for additional details on the different contributions to the bonding free energy). The expression for Δ thus encodes the salt, temperature and sequence dependence of the free-energy difference between bonded and non-bonded states. In general, the free energy computed according to the SantaLucia model does not take into account the effect of the presence of more than one non-binding nucleotide flanking the binding sequence [28]. Here the sticky ends are attached to the whole construct, which, particularly at low salt concentrations, might exert a substantial electrostatic repulsion that it is not straightforward to quantify in terms of contributions to the binding free energy. However, a recent joint numerical/experimental work provided a quantitative estimation of the effect of single-stranded tails on the melting temperature and hybridization free energy of short strands [32]. It was shown that the effect depends strongly on the salt concentration and seems to saturate when the length of the tail exceeds 4–5 single-stranded nucleotides. Unfortunately, the effect of double-stranded tails has not been investigated yet. In order to tentatively estimate the effect of the nanostar’s electrostatic repulsion on the phase behaviour, we use the long-tail limit of the values reported in [32] and extrapolate its dependence on *S* to correct the SantaLucia’s free energies. The detailed derivation of these additional terms is reported in Appendix A.

Once the free energy is known, the location of the critical point at a fixed salt concentration is found by invoking global stability; mathematically, the critical temperature $T_{c}$ and density $\rho_{c}$ must satisfy the conditions

(10) $$\frac{\partial P}{\partial \rho }{|}_{\left(T_{c},\rho_{c}\right)}=0\;\;\mathrm{and}\;\;\frac{\partial^{2}P}{\partial \rho^{2}}{|}_{\left(T_{c},\rho_{c}\right)}=0,$$

where $P\left(\rho ,T\right)=\rho^{2}\frac{\partial \bar{f}}{\partial \rho }$. We also evaluate the coexistence region by employing a standard common tangent construction [42]. We compute the pressure and chemical potential as

$$P=-\frac{\partial \bar{f}}{\partial v}=\rho^{2}\frac{\partial \bar{f}}{\partial \rho }\;\;\mathrm{and}\;\;\mu =g=\bar{f}+\frac{P}{\rho },$$

where *g* is the Gibbs free energy per particle, and we exploit the well-known property μ=g. We thus impose

(11) $$P_{1}\left(T,\rho_{1}\right)=P_{2}\left(T,\rho_{2}\right)\;\;\mathrm{and}\;\;\mu_{1}\left(T,\rho_{1}\right)=\mu_{2}\left(T,\rho_{2}\right),$$

where $P_{j}$ and $\mu_{j}$ are the pressure and chemical potentials in the phase *j*, respectively. In order to solve the system of equations given by Equations (10) and (11), we use a nonlinear solver (modified Powell algorithm) and set a grid of initial conditions in the ρ–*T* or $\rho_{1}$–$\rho_{2}$ plane at fixed *T*, for critical and coexistence points, respectively. Such a research strategy is proficient in finding critical and coexistence points.

## 3. Results

### 3.1. Structural Properties of the Fluid

We consider the reference fluid as a polymeric system composed of purely repulsive particles.

**Figure 2:**
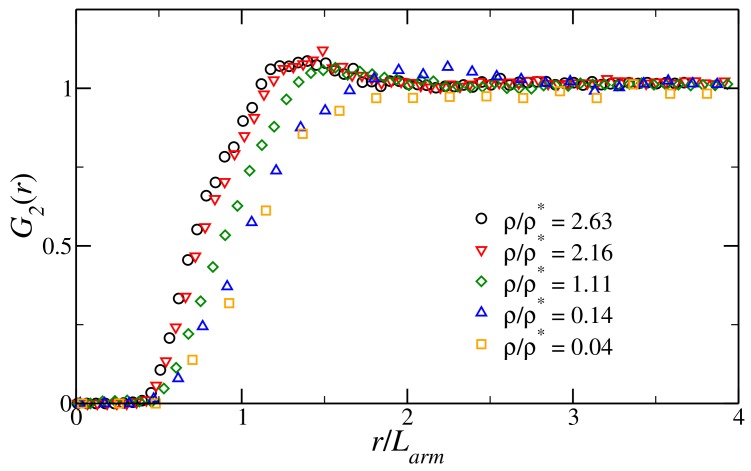
Radial distribution function of the centers of mass of the DNA tetramers as function of the inter-particle distance *r*, measured in units of the average arm length $L_{\mathrm{arm}}$, at fixed temperature T= 25 °C and salt concentration S= 0.5 M, for different reduced densities $\rho /\rho^{*}$.

We choose as the characteristic length scale of the fluid the length of one arm, defined as the average distance between the central nucleotides and any arm tip. This choice is dictated by the fact that the arms of the stars are quite rigid, particularly at a low salt concentration, as their length (≈20 nucleotides) is only a fraction of the double stranded (ds) DNA persistence length (≈150). On the contrary, the overall structure, as a result of the presence of the central unpaired nucleotides, can be very floppy [42]; thus the radius of gyration underestimates the size of the construct and does not provide a reliable measure for the typical interaction range. From numerical simulations, we find that $L_{\mathrm{arm}}$ depends on the salt concentration only, as we obtain $L_{\mathrm{arm}}=9.70$ nm for S=0.05 M, $L_{\mathrm{arm}}=8.93$ nm for S=0.2 M, and $L_{\mathrm{arm}}=8.72$ nm for S=0.5 M, regardless of temperature and functionality. It follows that the overlap density for our system, defined as $\rho^{*}=3/\left(4\pi L_{\mathrm{arm}}^{3}\right)$, is $\rho^{*}=2.61\times 10^{-4}$ nm${}^{-3}$ for S=0.05 M, $\rho^{*}=3.35\times 10^{-4}$ nm${}^{-3}$ for S=0.2 M and $\rho^{*}=3.60\times 10^{-4}$ nm${}^{-3}$ for S=0.5 M. We mention that, for the most dense systems considered, the arms become shorter and, overall, the nanostars start shrinking. This is usually a sign of the onset of the semi-dilute regime. Indeed, examining the radial distribution function $G_{2}\left(r\right)$, we note a structural change as $\rho /\rho^{*}≳$ 1. As is visible in Figure 2, for very low values of $\rho /\rho^{*}$, that is, in the dilute regime, the radial distribution function has no peak, the fluid having no structure. Upon approaching the overlap density, the fluid becomes more structured and a small peak appears. This peak happens at around $1.5L_{\mathrm{arm}}$, as is commonly observed in soft fluids at these densities [46]. These features support the choice of $L_{\mathrm{arm}}$ as the characteristic length scale of a nanostar in solution.

### 3.2. Pressure of the Reference Fluid

Next we analyze the pressure computed from the bulk simulations introduced in Section 2. As reported in the literature [47], the reduced (osmotic) pressure of semi-dilute solutions of polyelectrolytes with added salt should be a universal function of the reduced density $B_{2}\rho$, $B_{2}$ being the second virial coefficient.

**Figure 3:**
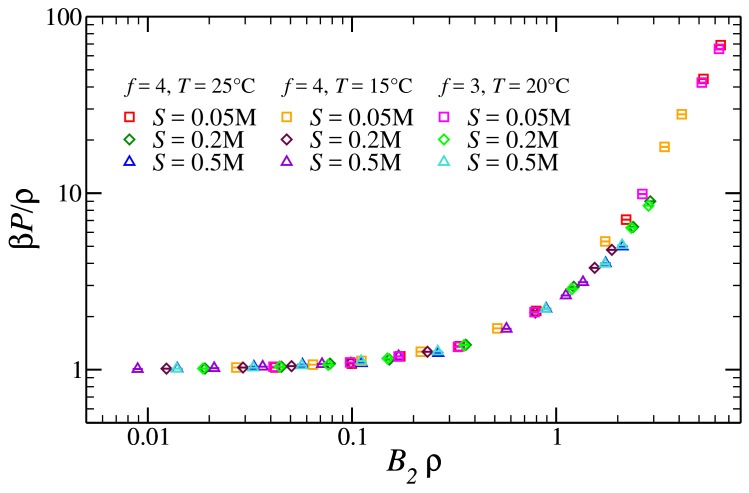
Reduced pressure for the reference fluid as function of the reduced density $B_{2}\rho$, for different functionalities, salt concentrations and temperatures.

In Figure 3, we report the reduced pressure βP/ρ as a function of the reduced density $B_{2}\rho$. We observe a nice collapse for both tetramers and trimers, at three different salt concentrations and three different temperatures. In what follows, we use these data to compute the reference free energy, using the two methods described in Section 2.

**Figure 4:**
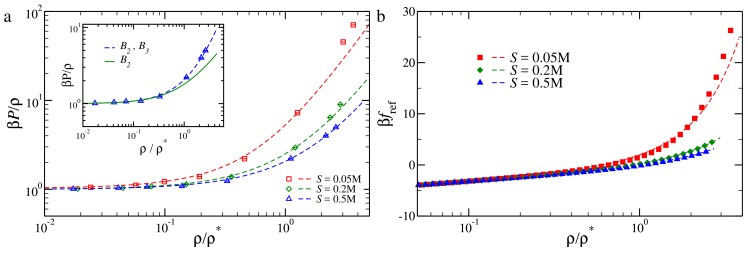
(**a**) Reduced pressure of a reference fluid of tetramers, as function of the reduced density $\rho /\rho^{*}$. Points are data from numerical simulations; dashed lines are polynomial fits (Equation (4)). Inset: Pressure as function of $\rho /\rho^{*}$ for S=0.5 M: symbols and dashed line as in the main panel; the full line is the second-order virial approximation; (**b**) Reference free energy as function of the reduced density $\rho /\rho^{*}$ computed using thermodynamic integration (TI) (full symbols) and a third-order virial approximation (dashed lines).

We start from the third-order virial approximation: panel (a) of Figure 4 shows the comparison between numerical data for the reduced pressure and the best fit of Equation (4) as a function of the reduced density. The dashed lines in Figure 4a refer to polynomial fits of Equation (4). In the fitting procedure, we fix $B_{2}$ to the values reported in [31]. The only fitting parameter is thus the third virial coefficient $B_{3}$: as shown in Figure 3, such a functional form fits the numerical data well for all the densities and salt concentrations considered; a small discrepancy is observed only for the lowest salt concentration S= 0.05 M, well in the semi-dilute regime. We further show, in the inset of Figure 4a, how the second-order virial approximation performs: the solid line is Equation (4), considered only up to the second order. The second-order approximation fits the data very nicely almost up to the semi-dilute regime, $\rho /\rho^{*}=1$, where the effective pair potential description for polymer solutions usually breaks down [48,49].

We further carry on the validation by comparing how the third-order virial approximation compares against thermodynamic integration. In Figure 4b, we present the reference free energy per particle $\beta f_{\mathrm{ref}}$ as a function of the reduced density, computed using the two methods: the third-order virial approximation agrees well with the TI, except, once again, at a high density for the lowest salt concentration. As shown in Section 3.3, the critical point always lies well within the dilute regime, where all the methods considered are bound to predict the same reference free energy. This strongly confirms the validity of the numerically undemanding approach adopted in [31].

### 3.3. Critical Points and Coexistence

We now compute the critical points using as excess free energy either the virial approximation (Equation (6)) or TI (Equation (3)).

**Figure 5:**
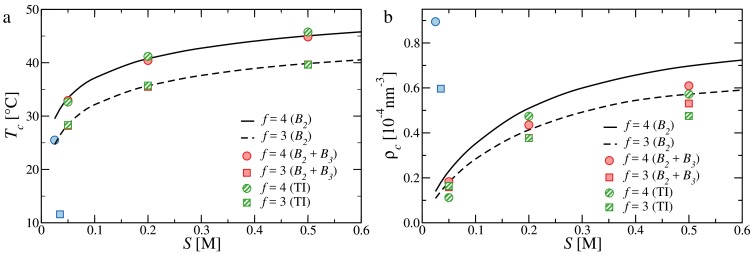
(**a**) Critical temperature as function of salt concentration. (**b**) Critical density as function of salt concentration. Circles and full line refer to tetramers (f= 4); squares and dashed line refer to trimers (f= 3). The data has been obtained with the second-order virial approximation [31] (black lines), the third-order virial approximation (full red symbols), thermodynamic integration (TI) (dashed green symbols) and experiments [8] (full blue symbols).

In Figure 5, we report critical temperatures (Figure 5a) and critical densities (Figure 5b) for tetramers and trimers. We also report the results of the second-order virial coefficient, as is done in [31], for direct comparison. We note that although we use the values of $B_{2}$ reported in [31], the two sets of results differ, as the corrections to ΔH and ΔS due to the presence of dangling ends were miscalculated (see Appendix A). Looking at the comparison, it is evident that the second-order virial expansion of the free energy is almost as accurate as the third-order and TI expressions in predicting the locus of the critical temperatures. Concerning the critical densities, the more accurate methods predict even smaller critical densities than the second-order virial approximation; the disagreement is, however, small with respect to the discrepancy that still persists with respect to the experimental data. Such a poor agreement should be ascribed to a limitation of the WTPT, which is known to underestimate the critical density [42,50].

**Figure 6:**
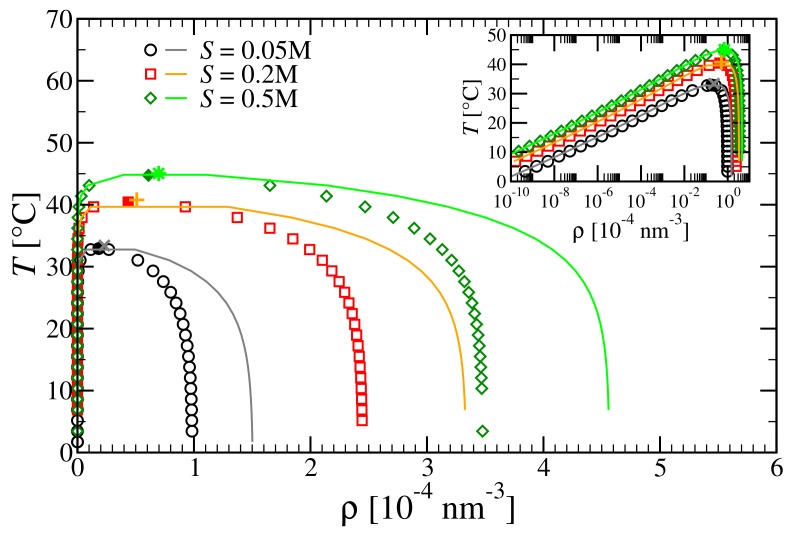
Main panel: Coexistence regions in the *T*–ρ plane for tetramers at different salt concentrations. Symbols and lines refer to data obtained with second-order virial approximation (full lines) and third-order virial approximation (open symbols); full symbols refer to critical points. Inset: As in main panel, semi-log scale emphasizes the low-density branch.

Next, we examine how employing more accurate expressions for the free energy affects the coexistence regions. In Figure 6, we report the prediction of WTPT for tetramers at different salt concentrations (trimers exhibit the same qualitative trends). The main panel, which shows a comparison between the temperature–density phase diagram computed with the second- (lines) and third-order (symbols) virial approximations, reflects the qualitative trends noted before for the critical point. As expected, the range of temperatures remains virtually unchanged, while the coexistence region shrinks towards smaller densities. From the inset of Figure 6, we can appreciate that the low-density branch remains untouched, as at low density the second-order virial expansion is already very accurate, while at higher densities the third term of the virial increases the excess free energy, thereby stabilizing the liquid.

**Figure 7:**
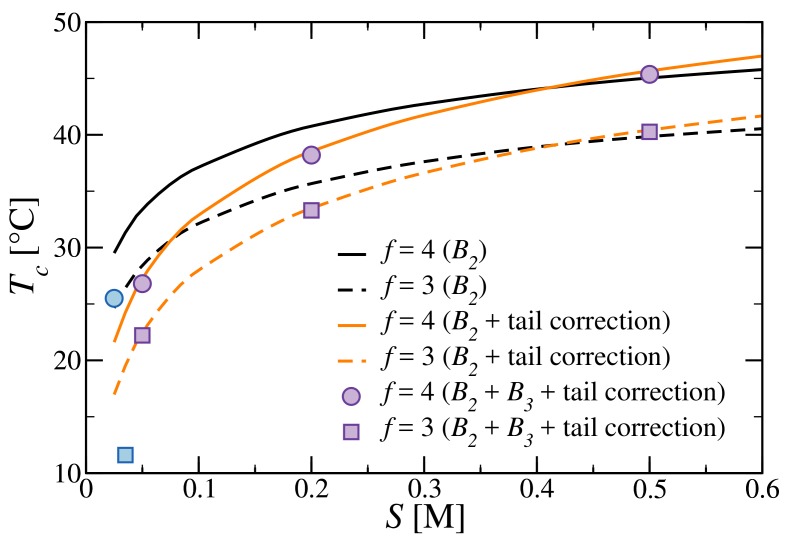
Critical temperature as function of salt concentrations, estimated by taking into account the contribution of the the repulsive tails, as is done in [32]. Circles and solid lines refer to tetramers (f=4); squares and dashed lines refer to trimers (f=3). The data has been obtained with the second-order virial approximation [31] (orange and black lines for data obtained with and without tail corrections), the third-order virial approximation (full violet symbols) and experiments [8] (full blue symbols). Data obtained with thermodynamic integration (TI) overlap with the $B_{2}+B_{3}$ symbols and are therefore omitted for clarity.

Lastly, we look at the effect of the tail correction of the free energy [32] on the critical temperature, as shown in Figure 7. The increased effective repulsion does not significantly change the critical density, which is therefore not shown. At a low salt concentration, the electrostatic repulsion shifts $T_{c}$ by as much as 8 K for both tetramers and trimers, improving the agreement with experimental results. Upon increasing *S*, the effect weakens and becomes effectively irrelevant above S≈0.3 M. The crossing of the lines obtained with and without corrections is most likely a spurious effect due to the salt dependence of the entropy change we assume (see Appendix A for details). Improving these estimates requires a better understanding of the tail effect on the DNA hybridization free energy for different secondary structures and local geometries, calling for additional experiments and simulations.

## 4. Conclusions

We have reported a computational study aimed at accurately evaluating the phase behaviour of all-DNA systems. This was done by employing the hybrid theoretical/numerical approach introduced in [31], where numerical simulations were used to calculate the inputs required by the WTPT to evaluate the system free energy. We performed extensive numerical simulations to precisely compute one of these inputs, namely, the reference free energy, for trivalent and tetravalent DNA nanostars at different temperatures, densities and salt concentrations. We have used this data to find a close comparison with the results obtained through a much simpler virial expansion truncated at the second order, as is done in [31].

The numerical results reported here show that such a second virial coefficient approximation faithfully reproduces the behaviour of the pressure in the reference fluid in a remarkably large interval of densities (essentially up to the semi-dilute regime). The agreement we observe demonstrates that, indeed, the effective pair-wise interaction is sufficient to reproduce the reference fluid structure and provides an inexpensive and yet accurate route to estimate the pressure, even at a much higher density. Next, we have used the pressure data to estimate the reference free energy necessary for WTPT by truncating the virial expansion to the third term and by performing TI. The critical points computed with the WTPT exhibit a good agreement with each other and with the much simpler second-order virial approximation, once again demonstrating the validity of the approach developed in [31]. The only notable difference is a shift of the density of the coexisting liquid towards smaller values, which is to be ascribed to the increased repulsion with respect to the second-order virial expansion.

We have also looked at the effect of including an additional correction to the free energy because of the presence of nearby non-binding nucleotides on the hybridization of the sticky ends, estimated by extrapolating values taken by [32]. This additional contribution effectively reduces the critical temperature, improving the agreement with experiments [8]. As noticed, the presence of the tails affects the position of the critical points at low salt concentrations, while, upon increasing *S*, the effect weakens and becomes irrelevant above S≈0.3 M. This is consistent with the electrostatic origin of the free energy shift, as reported in [32].

To conclude, we have shown that the method developed in [31] is not only very accurate despite its simplicity, but is also easily extensible by incorporating additional terms into the WTPT’s theoretical description of the system. The agreement between the theoretical and experimental results shows that this approach already allows for a simple yet accurate in silico evaluation of the phase behaviour of all-DNA systems. The flexibility of the method, which is not tied to the specific DNA sequences or geometry of the nanoconstructs, makes it a powerful tool to assist future experimental realizations of all-DNA materials [1].

## Appendix A. Calculation of the Hybridization Free Energy

In general, the hybridization free energy at any temperature is given by

(A1) ΔG(T)=ΔH+TΔS,

where ΔH and ΔS are the difference in enthalpy and entropy, which are assumed to be *T*-independent. From SantaLucia, the free energy at 37 degrees Celsius is given by

(A2) $$\begin{array}{cc} \Delta G(37^{\circ }) & =\Delta G_{\mathrm{init}}\left(37\;{}^{\circ}C\right)+\Delta G_{\mathrm{sym}}\left(37\;{}^{\circ}C\right)+\sum \Delta G_{\mathrm{stack}}\left(37\;{}^{\circ}C\right)+\Delta G_{\mathrm{term}}\left(37\;{}^{\circ}C\right)+\Delta G_{\mathrm{dangl}}\left(37\;{}^{\circ}C\right). \end{array}$$

The sequence we are interested in is $\left(5^{\prime }A\right)-CGATCG-3^{\prime }$: this duplex is self-complementary, which means $\Delta G_{\mathrm{symmetry}}\left(37\;{}^{\circ}C\right)\neq 0$, and is attached to the arm of the nanostar through an unpaired *A* base at the $5^{\prime }$ end.

$$\begin{array}{c} (5^{\prime }A)-CGATCG-3^{\prime }=\Delta G_{\mathrm{sym}}\left(37\;{}^{\circ}C\right)+CG+GA+AT+TC+CG+\Delta G_{\mathrm{term}}\left(37\;{}^{\circ}C\right) \end{array}$$

(A3) $$\begin{array}{c} 3-GCTAGC-(A5^{\prime })\;\;\;\;\;GC\;\;\;CT\;\;\;TA\;AG\;\;GC \end{array}$$

plus the additional contribution due to the dangling ends. SantaLucia gives the values for ΔH and ΔG(37 °C) only. The entropy difference can be estimated by using Equation (A1), as

(A4) $$\Delta S=\frac{\Delta H-\Delta G}{T},$$

from which, at *T* = 37 C and S= 1 M, we can obtain the entropy difference ΔS.

Table A1 reports the SantaLucia contributions due to the stacking; the entropy difference has been estimated by using Equation (A4). We note that the $5^{\prime }$ and $3^{\prime }$ ends are reversed with respect to the convention used in oxDNA. Data reported in Table A1 are considered accordingly.

**Table A1:** Hybridization enthalpies ΔH, entropies ΔS and free energies ΔG at T= 37 °C for the sequence $\left(5^{\prime }A\right)-CGATCG-3^{\prime }$ according to SantaLucia nearest-neighbor DNA model

Sequence	ΔH (kcal/mol)	ΔS (cal/K·mol)	ΔG(37 °C) (kcal/mol)
CG/GC	−9.8	−24.38	−2.24
GA/CT	−7.8	−21.02	−1.28
AT/TA	−7.2	−21.34	−0.58
TC/AG	−7.8	−21.02	−1.28
CG/GC	−9.8	−24.38	−2.24

The sum of all stacking contributions are

$\Delta H_{\mathrm{stack}}=-42.4$ kcal/mol $\Delta S_{\mathrm{stack}}=-112.139$ cal/K·mol.

The contribution for the initial G/C pair amounts to

$\Delta H_{\mathrm{init}}=0.2$ kcal/mol $\Delta S_{\mathrm{init}}=-5.7$ cal/K·mol.

The symmetry correction carries a contribution

$\Delta H_{\mathrm{sym}}=0$ kcal/mol $\Delta S_{\mathrm{sym}}=-1.4$ cal/K·mol.

Before the dangling end correction, the total enthalpy and entropy differences are ΔH=−42.2 kcal/mol and ΔS=−119.23 cal/K·mol. Finally, following SantaLucia, the sticky end sequence has two CA/G $3^{\prime }$ dangling ends. Each dangling end gives a contribution of

$\Delta H_{\mathrm{dangl}}=-5.9$ kcal/mol $\Delta S_{\mathrm{dangl}}=-16.38$ cal/K·mol.

The final free energy difference is therefore ΔH=−54 kcal/mol and ΔS=−151.99 cal/K·mol.

We complement the calculation of the bonding free energy by also taking into account the effect of the nanostar on the bond formation, following [32]. As reported in the paper, the shift in the hybridization free energy δΔG observed experimentally, due essentially to electrostatic repulsions, is independent of the duplex length, depends on the salt concentration *S* and reaches a constant value for sufficiently long tails ($n_{\mathrm{tail}}≳4$). Because the “tail” is, in our case, an entire double-stranded arm composed of 10 nucleotides, we consider the limiting (plateau) value of δΔG at the proper salt concentration. We then consider Section S3.3 and Figure S7 of the Supplementary Information of [32], in which a numerical estimate for the extra contribution to the hybridization enthalpy and entropy in the presence of inert tails is provided. The data reported concern a duplex of length $n_{\mathrm{hybrid}}=9$ with tails of length $n_{\mathrm{tail}}=7$, and hence these numbers should be taken with a grain of salt. Rescaling the numerical δΔG to the experimental values (at fixed salt concentration S=0.05 M) and rescaling the enthalpy and entropy shifts accordingly, we obtain that

(A5) $$\delta \Delta H_{\mathrm{tail}}=0.30296\;\mathrm{kcal}/\mathrm{mol}\;\;\delta \Delta S_{\mathrm{tail}}=-0.00260\;\mathrm{kcal}/K\cdot \mathrm{mol}.$$

We assume that $\delta \Delta S_{\mathrm{tail}}=\delta \Delta S_{\mathrm{tail},0}$ + αln(S); such a choice is motivated by the observed dependence of $\delta \Delta G_{\mathrm{tail}}$ on the salt concentration, which we assume, following SantaLucia and for the lack of further experimental data, to have a purely entropic origin. Fitting the experimental data (Figure 3e of [32]), we obtain α=1.6821 cal/K and $\delta \Delta S_{\mathrm{tail},0}=2.4525$ cal/K. The shift in the Gibbs free energy for every temperature and every salt concentration is then given by

(A6) $$\delta \Delta G_{\mathrm{tail}}=\delta \Delta H_{\mathrm{tail}}+\left(\delta \Delta S_{\mathrm{tail},0}+\alpha \ln\left(S\right)\right)T.$$
